# Deciphering Spatial Protein–Protein Interactions in Brain Using Proximity Labeling

**DOI:** 10.1016/j.mcpro.2022.100422

**Published:** 2022-10-02

**Authors:** Boby Mathew, Shveta Bathla, Kenneth R. Williams, Angus C. Nairn

**Affiliations:** 1 Yale/NIDA Neuroproteomics Center, New Haven, Connecticut, USA; 2 Molecular Biophysics and Biochemistry, Yale University School of Medicine, New Haven, Connecticut, USA; 3 Department of Psychiatry, Yale University, New Haven, Connecticut, USA

**Keywords:** protein–protein interactions, proximity labeling, biotinylation, neuroproteomics, protein interaction network, AAV, adeno-associated virus, AD, Alzheimer’s disease, AIS, axon initial segment, Alk, anaplastic lymphoma kinase, ALS, amyotrophic lateral sclerosis, AP, affinity purification, APEX2, ascorbate peroxidase 2, BACE1, beta-site amyloid precursor protein cleaving enzyme 1, BAR, biotinylation by antibody recognition, BASU, *Bacillus subtilis*–derived biotin ligase, BMAL1, Brain and Muscle ARNT-Like 1, CNS, central nervous system, co-IP, coimmunoprecipitation, CRM, chromatin regulatory module, CSF, cerebrospinal fluid, CTF, C-terminal fragment, DAT, dopamine transporter, Eph, ephrin, ER, endoplasmic reticulum, FDR, false discovery rate, FTD, frontotemporal dementia disease, HD-PTP, His domain phosphotyrosine phosphatase, HEK293, human embryonic kidney 293 cell line, H_2_O_2_, hydrogen peroxide, HRP, horseradish peroxidase, iBioID, *in vivo* BioID, LAMP1, lysosomal-associated membrane protein 1, MeCP2, methyl-CpG binding protein 2, MS, mass spectrometry, NCC, neural crest cell, NDD, neurodevelopmental disorder, NES, nuclear export sequence, NF186, neurofascin-186, NPC, nuclear pore complex, PARP6, poly-(ADP-ribose) polymerase 6, PD, Parkinson’s disease, PL, proximity labeling, PN, projection neuron, polyQ, polyglutamine, PPI, protein–protein interaction, PPIN, PPI network, PTM, post-translational modification, RGC, retinal ganglion cell, RTT, Rett syndrome, SC, superior colliculus, SCA1, spinocerebellar ataxia type 1, SPN, spiny projection neuron, SubMAPP, subcellular-specific uncaging-assisted biotinylation and mapping of phosphoproteome, α-syn, alpha-synuclein, TADA2a, transcriptional adapter 2-alpha, TDP-43, TAR DNA-binding protein-43, TLK2, Tousled-like kinase 2, TWIST1, Twist Family BHLH Transcription Factor 1, VM, ventral midbrain, Y2H, yeast two-hybrid

## Abstract

Cellular biomolecular complexes including protein–protein, protein–RNA, and protein–DNA interactions regulate and execute most biological functions. In particular in brain, protein–protein interactions (PPIs) mediate or regulate virtually all nerve cell functions, such as neurotransmission, cell–cell communication, neurogenesis, synaptogenesis, and synaptic plasticity. Perturbations of PPIs in specific subsets of neurons and glia are thought to underly a majority of neurobiological disorders. Therefore, understanding biological functions at a cellular level requires a reasonably complete catalog of all physical interactions between proteins. An enzyme-catalyzed method to biotinylate proximal interacting proteins within 10 to 300 nm of each other is being increasingly used to characterize the spatiotemporal features of complex PPIs in brain. Thus, proximity labeling has emerged recently as a powerful tool to identify proteomes in distinct cell types in brain as well as proteomes and PPIs in structures difficult to isolate, such as the synaptic cleft, axonal projections, or astrocyte–neuron junctions. In this review, we summarize recent advances in proximity labeling methods and their application to neurobiology.

## Highlights

• Exploring PPIs in brain is essential to understanding neurological disorders.
• Overview of current PL methods used for PPI identification.
• Application of *in vitro* and *in vivo* PL approaches in neurobiology.
• Future perspectives of PL methods in neurobiology.

## Graphical Abstract

**Figure:**
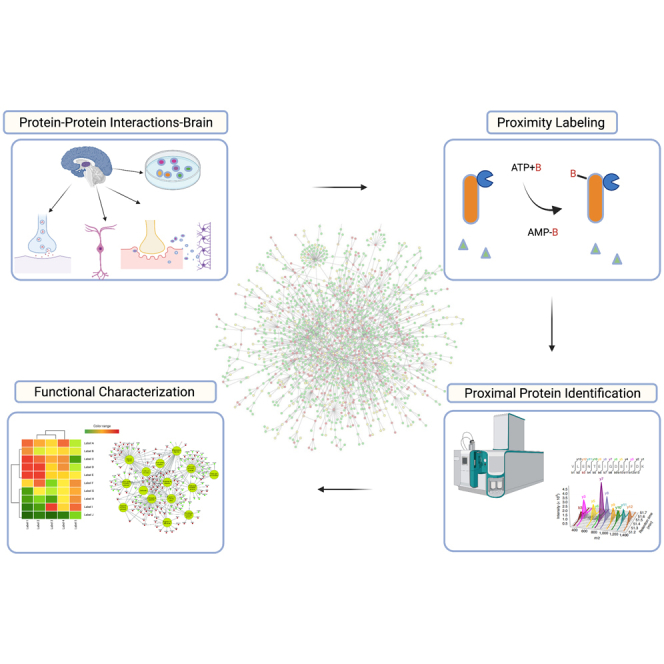

## In Brief

PL has emerged as a powerful tool to identify proteomes in distinct cell types in brain as well as proteomes and protein–protein interaction networks in structures difficult to isolate, such as the synaptic cleft, axonal projections, or astrocyte–neuron junctions. Here, we review recent advances in PL methods and their application to neurobiology.

Cellular functions in eukaryotes are dependent upon and are tightly regulated by biomolecular interactions between proteins and small molecules, nucleic acids, and other proteins. Indeed, eukaryotic proteins are rarely found as monomers, rather, they almost always are assembled into multiprotein complexes (1, 2). A reference map of the human binary protein interactome based on yeast two-hybrid (Y2H) screening contains 52,569 verified protein–protein interactions (PPIs) involving 8275 proteins (3), the latter corresponding to about 43% of the 19,116 human protein-coding genes (4). The close physical location of proteins and their domains within these complexes confers their functions and signaling properties. The elucidation of interactome networks is, therefore, one of the most efficient methods to understand normal cellular processes at the molecular level (5). Since the natural patterns of PPIs are disrupted when the homeostatic condition of an organism or an individual cell is disturbed as a result of environmental stress or disease (6), identification of spatially resolved protein-binding partners will help to decipher protein functionality and the associated protein pathways.

A prime illustration of the critical roles of PPIs is found in the central nervous system (CNS). For example, a survey of 60 synaptic proteins showed that all were assembled into complexes involving >200 other proteins (7). Disruption of synaptic signaling complexes, as evidenced by mutations in the postsynaptic scaffold protein PSD95 and its interacting proteins, causes behavioral abnormalities and interferes with synaptic plasticity (8, 9, 10). Given the importance of protein complexes to neural signaling and synaptic plasticity, mapping the brain protein interactome(s) is therefore key to understanding its functions.

The complexity of brain functions is dependent upon the temporal and spatial control of differentially expressed proteins and their associated specific interactors in perhaps as many as 1000 different brain cell types. Exploration of PPI network (PPIN) variations in a cell type–specific manner should provide valuable information to help understand the brain's complexity and pathogenesis. In the last decade, an innovative approach called proximity labeling (PL), most frequently using biotinylation, that can spatially resolve the PPIs of specific target proteins *in vivo* or conditions approaching *in vivo*, has gained considerable interest. This review first briefly discusses the various PL approaches most often used to identify and interpret protein networks (see references for more in-depth reviews) (11, 12, 13, 14). We then focus on the different types of PL approaches used in the field of neurobiology to identify PPIs in brain, highlighting its application to understanding the role of PPIs in the underlying pathophysiological mechanisms of neurobiological disorders.

## PL—General Principles

Several *in vitro* methods such as affinity purification (AP), coimmunoprecipitation (co-IP), protein crosslinking, and the Y2H approach, in many cases coupled to mass spectrometry (MS)–based proteomics, have been developed to identify PPIs in various biological systems (15, 16, 17). AP–MS has also been used in conjunction with nucleic acid sequencing techniques, such as chromatin immunoprecipitation sequencing (18) and RNA immunoprecipitation sequencing (19), to identify protein–nucleic acid interactions. Although AP–MS is a powerful method to capture higher affinity protein interactors, nonspecific binding and the inability to detect transient interactions are major drawbacks (20). The Y2H system can give an efficient and quantitative estimation of binding affinities down to at least a *K*_*D*_ of approximately 25 μM (21). A more recent approach, next-generation Y2H system (Y2H-next generation interaction screening), uses deep sequencing to identify candidate interactors (22). Despite the methodological advantages of Y2H-next generation interaction screening, the complex datasets resulting from this technology pose unique bioinformatic and statistical challenges for analysis (23).

PL or tagging of proteins provides a viable approach for characterizing strong as well as transiently associated protein complexes that may be held together by relatively weak interactions and extended protein clusters where not all proteins in the same multiprotein complex interact directly with each other. The latter is important as the overall proximity of proteins in a network rather than just the immediate binding partners determines their biological functions (24). In addition, PL provides a means to identify PPIs within specific cell types or in spatially confined intracellular compartments. For all these reasons, PL coupled to MS is now widely used as a technology of choice to explore PPIs (25, 26). In general, PL works by expressing a chimeric assembly of the protein of interest and a modifying enzyme, enabling enzyme-catalyzed conversion of a substrate into a highly reactive intermediate that is conjugated with an affinity tag such as biotin. These activated substrates diffuse and covalently label nearby endogenous proteins in a proximity-dependent manner. The proteins proximal to the enzyme are more likely than distal ones to be labeled as labeling efficiency depends on local density of intermediate and target protein and drops off as a function of distance from the modifying enzyme (27). Since the yield of labeled protein or peptide in any PL study is influenced by many factors (*e.g.*, local protein concentration, number and distribution of exposed modifiable residues, digested peptide yield, peptide ionization, and fragmentation), relating affinity and yield is not straightforward. The labeled proteins, or peptides derived from digested proteins, are enriched using affinity-based approaches (*e.g.*, streptavidin or antibiotin antibody) prior to their subsequent identification and characterization (28). As a recent example of the power and scope of the approach, a biotinylation-based map of 192 subcellular markers revealed the intracellular locations of 4145 unique proteins in human embryonic kidney 293 (HEK293) cells and enabled the identification of proteins at the interface between the mitochondrial outer membrane and the ER that are crucial for mitochondrial homeostasis (29).

### PL Enzymes

**Fig. 1:**
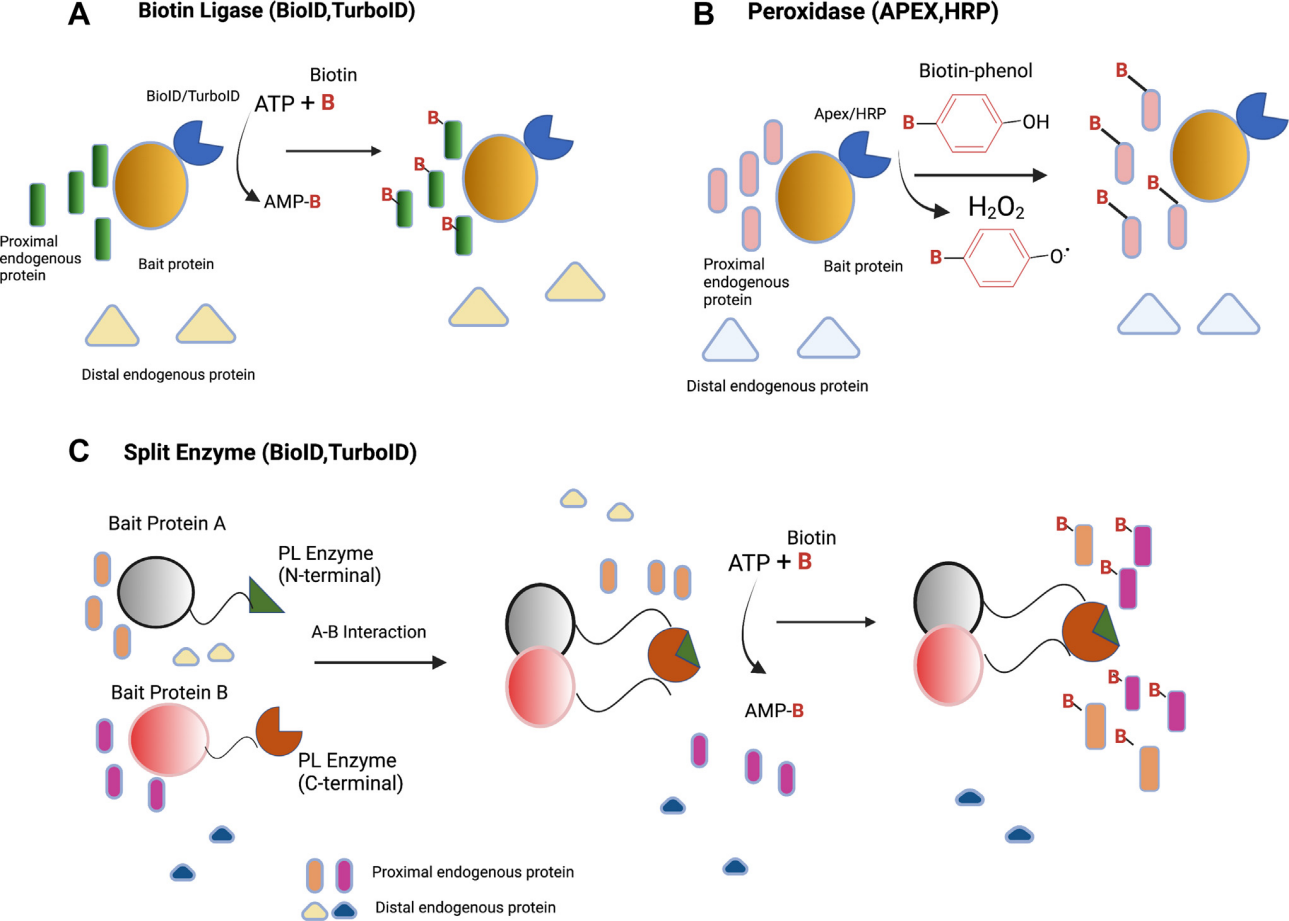
**Biotin ligase, peroxidase, and split-based PL methods are most commonly used for PPI mapping in neuroscience.***A*, biotin ligase–based approaches, such as BioID or TurboID, utilize ATP and biotin to catalyze the formation of reactive biotin-5′-AMP that diffuses and labels proximal proteins. *B*, peroxidase-based approaches, such as APEX or HRP, use hydrogen peroxide to oxidize biotin–phenol into reactive phenoxyl radicals that preferentially label proximal over distal endogenous proteins. *C*, the split approach consists of N-terminal and C-terminal fragments of a PL enzyme that are driven together by a PPI or membrane–membrane apposition to reconstitute an active enzyme. APEX, ascorbate peroxidase; HRP, horseradish peroxidase; PL, proximity labeling; PPI, protein–protein interaction.

PL uses engineered enzymes (Table 1) such as peroxidases (ascorbate peroxidase 2 [APEX2] (30), horseradish peroxidase [HRP]) (31), or biotin ligases (BioID (32, 33), BioID2 (34), *Bacillus subtilis*–derived biotin ligase (BASU) (35), TurboID, and miniTurbo (36)) to map PPIs. The two most commonly used PL enzymes are the BirA mutant (BioID, proximity-dependent biotin identification) and APEX. Figure 1 shows schematic representations of the modes of labeling of commonly used PL enzymes. Table 2 summarizes the general applications of PL and the strengths and limitations of the different PL enzymes.

**Table 1:** PL enzymes commonly used for PPI studies

Enzyme	Origin	Size (kDa)	Optimal temp (°C)	Labeling time	Labeling radius (nm)	Substrates and cytotoxicity	Model organisms
APEX	Pea	28	37	1 min	<20	Biotin–phenol + H_2_O_2_ (toxic)	Mammalian cells, flies
APEX2	Soybean						Mammalian cells, bacteria, *Chlamydia*, yeast
Split-APEX2						Biotin–phenol + H_2_O_2_ (toxic) + heme	Mammalian cells, yeast
HRP	Horseradish	44	37	≤1 min	200–300	Biotin–phenol + H_2_O_2_ (toxic)	Human and chicken cells
Split-HRP						Biotin–phenol + H_2_O_2_ (toxic) + heme	Mammalian, yeast cells
BioID	*E. coli*	35	37	18–24 h	∼10	Biotin (nontoxic)	Mammalian, plant cells, yeast
BioID2	*A. aeolicus*	25		18 h			Mammalian, plant cells
Split-BioID							Mammalian, plant cells
BASU	*B. subtilis*			1 min			Mammalian, plant cells
AirID	*E. coli*	37	26	3–24 h			Mammalian cells and wheat cell–free system
miniTurboID		28	25	≤10 min			Mammalian, plant cells, yeast, flies, and worms
TurboID							
Split-TurboID		35		1 h			Mammalian cells

**Table 2:** Comparison between different PL approaches

Enzyme	Organelles	Strengths	Limitations
APEX2	Cytosol, nucleus (138), mitochondrial matrix (139), mitochondrial intermembrane space (48), mitochondrial nucleoid (139), ER lumen (140), ERM (141), OMM (142), Golgi (143), autophagosome (144), and cilia (145)	High temporal resolution and high activity in most cellular compartments. Can be used for RNA labeling and electron microscopy	Limited application *in vivo* because of H_2_O_2_ toxicity and low permeability
HRP	ER lumen, cell surface (146), and synaptic cleft (54)	Higher activity than APEX2 in the secretory pathway. Used for cell surface proteomics	Limited to secretory pathway and extracellularly; limited use *in vivo* because of H_2_O_2_ toxicity and low permeability
BioID	Cytosol, nucleus (147), ERM (148), mitochondrial matrix (149), mitochondrial outer membrane (150), mitochondrial nucleoid (151), cell surface (152), peroxisome membrane (153), Golgi (154), and cilia (155)	Nontoxic labeling conditions and ideal for most cellular compartments	Poor temporal resolution and limited application i*n vivo* because of low catalytic activity
BioID2		Higher activity than BioID. Stable at higher temperatures	
BASU	Cytosol, nucleus (156)	Higher activity than BioID	
AirID	Cytosol, nucleus (46)	Higher activity than BioID. Lower potential toxicity in long-term experiments	
TurboID	Cytosol, nucleus, ERM, ER lumen, mitochondrial matrix, and cell surface (36)	Highest activity promiscuous biotin ligase	Less user control of labeling window because of high biotin affinity. Toxicity in long-term experiments
miniTurboID		High resolution and tight control of user-defined labeling window	Lower catalytic activity and stability compared with TurboID
Split-APEX2	Cytosol, nucleus, ER–mitochondria contact sites, and cell surface (157)	High reconstituted activity	Limited application *in vivo* because of reagent toxicity and low reagent permeability; requires supplementation of heme cofactor
Split-HRP	ER lumen, cell surface, and neuronal synapse (95)	High reconstituted activity	Limited application *in vivo* because of reagent toxicity and low reagent permeability; requires supplementation of heme cofactor; limited to secretory pathway and extracellular applications
Split-BioID	Cytosol, nucleus, and ER–mitochondria contact sites (57, 58, 59)	Nontoxic labeling conditions	Poor temporal resolution and very low catalytic activity

### Biotin Ligase–Based PL

The PL methodology developed by Parrott and Barry (37, 38) used the mammalian endogenous biotin ligase to biotinylate recombinant proteins that contained a biotin acceptor tag. BirA uses ATP and biotin to produce reactive biotin-5ʹ-AMP that is held within the BirA active site until it reacts specifically with lysine residues of the biotin acceptor tag sequence in its substrate proteins, which are primarily restricted to a few carboxylases in mammalian cells (39). Since WT BirA exhibits limited substrate specificity, Roux *et al.* (32) generated a more promiscuous enzyme, termed BirA∗, which has a catalytic site mutation (R118G), which reduces affinity for biotin-5′-AMP so that it can diffuse from the ligase and react with free primary amines of exposed lysine residues on neighboring proteins within an approximate 10 nm range (32, 40) (Fig. 1*A*). The large size of the 35.1 kDa BirA∗ prevents efficient targeting of some fusion proteins (34), and it restricts passage through nuclear pore complexes (NPCs) of integral inner nuclear membrane proteins (41). This challenge can be circumvented to some extent by using a smaller 26.6-kDa second-generation BioID2 from *Aquifex aeolicus*. Advantages of BioID2 include improved localization, increased biotin sensitivity, and use at temperatures >37 °C (34). Nonetheless, there are shortcomings with Bio-ID and BioID2 because of their slow kinetics that necessitates labeling with biotin for 18 to 24 h (36). This limits use of BioID and BioID2 for studying dynamic processes that occur on the time scale of minutes or even a few hours (36).

To address the kinetic limitations, an engineered 28 kDa BASU was generated that dramatically reduces the labeling time to 1 min without toxicity to living cells (35). Similarly, Branon *et al.* (36) used yeast display–based directed evolution to engineer two promiscuous mutants of *Escherichia coli* biotin ligase, termed TurboID and mini-Turbo, which effectively biotinylate proteins in 10 min or less in cell culture upon biotin addition. TurboID and mini-Turbo also retain catalytic activity at lower temperatures (*T* < 37 °C) with TurboID being approximately twofold more active than mini-Turbo (42, 43). The TurboID ligase has been used to characterize protein complexes in several cell compartments (*e.g.*, nucleus, mitochondrial matrix, endoplasmic reticulum [ER] lumen, ER membrane) and organisms, such as flies (44), yeast (45), and plants (42). Very recently, Air-ID was developed that showed more specific tagging of interaction partners and was less toxic to cells over long incubation periods (46). An *in vitro* BioID labeling method also has been developed recently that allows instantaneous labeling of protein from lysed cells (47).

### APEX-Based PL

Initially, APEX was developed to catalyze the hydrogen peroxide (H_2_O_2_)-dependent polymerization of diaminobenzidine that is used to generate high-resolution electron microscopy images, and it was then further optimized for proximity-dependent protein biotinylation (30, 48, 49). In the presence of H_2_O_2_, APEX oxidizes biotin–phenols to phenoxyl radicals that biotinylate nearby proteins on the side chains of electron-rich amino acids, such as cysteine, histidine, tyrosine, and tryptophan (Fig. 1*B*). Since the resulting biotin phenoxyl radical has a short half-life of <1 ms, the biotinylation efficiency dramatically decreases beyond >20 nm of the APEX active site (50). An improved second-generation enzyme, APEX2, was engineered by yeast display–directed selection that is far more active in cells and that provides improved enrichment of endogenous mitochondrial and ER membrane proteins (30). The A134P mutation in APEX2 confers improvements in kinetics, thermal stability, heme binding, and, most strikingly, resistance to inhibition by high H_2_O_2_ concentrations (27). Only phenolic compounds had been generally used as APEX2 substrates, limiting application of APEX2 to labeling of electron-rich amino acids in proteins. Zhou *et al.* (51) identified biotin-conjugated arylamines as novel probes with significantly higher reactivity toward nucleic acids and captured all 13 mitochondrial mRNAs in the mitochondrial matrix and none of the cytoplasmic RNAs. APEX2-mediated labeling of RNA thus provides a promising method for mapping the subcellular transcriptome (51).

### HRP-Based PL

HRP is a peroxidase that in the presence of H_2_O_2_ is able to convert a substrate into a highly reactive radical that covalently tags neighboring proteins on the aforementioned electron-rich amino acids (52). Since the HRP structure is dependent upon two Ca^2+^ ion-binding sites and four disulfide bonds that are broken in the reducing conditions that exist within cells (*e.g.*, in the cytosol), HRP-based PL is limited to use in oxidizing environments, such as the lumen of the ER or the Golgi (53), and extracellular applications such as mapping protein interactomes at the cell surface (54). The selective proteomic PL assay using tyramide and enzyme-mediated activation of radical sources are HRP-based PL techniques specifically used for the identification of membrane-bound protein clusters and cell surface complexes, respectively (55, 56).

### Split-PL

Split-PL systems are useful for studying additional interacting components of spatiotemporally defined binary protein complexes and membrane contact sites. PL enzymes such as HRP, APEX2, BioID, or TurboID are split into two modular parts that are reconstituted into a functional entity when in close proximity (Fig. 1*C*). HRP was the first enzyme used for the development of the split-PL system; however, the need for H_2_O_2_ and heme for activity of the split forms of HRP and APEX2 limits their utility for *in vivo* studies (28). BioID can be split into two parts at two different amino acid sites: E140/Q141 and E256/G257. Comparative analysis showed that the E256/G257 version exhibited stronger biotinylating activity (57, 58). Recently, Kwak *et al.* (59) developed the Contact-ID method, which splits BioID between amino acid residues G78/G79 and which has higher biotinylation activity than the split-BioID pair cleaved at the E256/G257 site. A version of TurboID, which is split at amino acid site L73/G74, has also been used to profile the composition of ER–mitochondria contact sites (60). The Contact-ID proteome was more biased toward ER–mitochondria proteins, whereas the split-TurboID approach showed more balance between the identified outer mitochondrial membrane and ER membrane proteins. This difference may arise from the decreased labeling time required for split-TurboID (4 h) as compared with Contact-ID (16 h).

### Biotinylation by Antibody Recognition

The biotinylation by antibody recognition (BAR) method is designed to identify proteins in the vicinity of an antibody in a complex with antigen *in situ*. For labeling, a fixed and permeabilized tissue sample is incubated with a primary antibody to target a protein of interest. In the presence of H_2_O_2_ and biotin–phenol, a secondary HRP-conjugated antibody creates free radicals that result in biotinylation of proteins in close proximity to the target protein. Streptavidin-coated beads are used to pull down the biotinylated proteins that are then identified by MS (61). In a variation of the BAR method, Barriopedro *et al.* (62) have recently developed a recombinant ProtA-Turbo enzyme that acts as an “off the shelf” proximity biotinylation enzyme. This approach facilitates interaction proteomics studies in fixed and nonfixed primary cells or clinical samples.

## MS Data Analysis

Significant progress in the application of PL approaches requires MS-based quantitative proteomics to provide comprehensive identification of PPIs related to the protein of interest. Multiple MS approaches can be used to discriminate the enrichment of proteins in experimental conditions as compared with negative controls. In addition to label-free approaches based on spectral counting or MS1 intensity/area under the curve (14, 63), isobaric labeling approaches also provide powerful technologies for PL-based enrichment experiments. Hence, Cho *et al.* (28) used tandem mass tag–based quantitation for mapping PPIs and subcellular proteomes in live mammalian cells. The resulting peptide MS/MS spectra are processed for protein identification through the use of several platforms (14, 32, 42, 64).

Since bait–prey based PPI analyses typically retrieve nonspecific interactors that copurify with the protein(s) of interest, it is necessary to carry out appropriate controls such as use of nonchimeric PL enzyme to help differentiate specific from nonspecific interactions (65). Additional controls such as an unrelated protein carrying the same tag or the tag alone also can be included. For example, GFP, which is unlikely to interact with many proteins, can be used as a control bait. Identified interactors in control samples are presumably false positives because of the epitope tags or the affinity capture method (15). It is also important to appreciate that identification of larger numbers of protein “hits” in an interactome dataset does not necessarily represent more true positive proteins. The implementation of appropriate controls, stringent false discovery rates, and other appropriate filters is essential to identify real PPIs. In this regard, implementing a quantitative filter enables high-confidence detection of specific interactors amongst a vast excess of background proteins (66). Specifically, several bioinformatic approaches have been developed to help differentiate specific from nonspecific PPIs (15). A score can be assigned to each PPI that seeks to quantify relative confidence of the identified interaction by combining several quantifiable parameters, such as reproducibility, specificity, and the relative abundance of each identified protein. Scoring algorithms include MiST (67), CompPASS (68), SAINT (69), and CRAPome (70). While SAINT uses quality controls and quantitative data for a given prey to determine the probability that an interaction between the prey and bait protein is a true positive, CompPASS utilizes several scoring parameters that ultimately focus on abundance, uniqueness, and reproducibility to distinguish between true interactors and contaminating background proteins. High-scoring interactors can then be imported into STRING (71) and Cytoscape software for visualizing, modeling, and analyzing protein networks (72). Various software can be used for functional annotation and enriched pathway analysis of interactomes (73, 74, 75, 76).

## *In Vivo* Applications of BioID

Important questions with regard to *in vivo* BioID (iBioID) studies in mice are the optimum method for biotin administration and the tissue availability of administered biotin. For example, although intraperitoneal biotin injection has been used in mice (77), it has the physical burden of repeated injections for 3 to 7 days. In addition, to obtain the high concentration needed for injection, biotin must be dissolved in a solution containing an organic solvent, such as dimethyl sulfoxide (78). Since dimethyl sulfoxide treatment induces biological effects (79, 80), this route of administration may alter the PPIs being studied. Since biotin supplementation to drinking water (3.7 mg/ml) failed to induce protein biotinylation in a study in titin-BirA∗ knock-in mice, it is possible that this route of administration is not sufficient for efficient biotinylation (81). That is, the biotin dose is restricted because of the low solubility of biotin in water. As a result, Murata *et al.* (82) examined the effect of biotin feeding on PL in several tissues in a Brain and Muscle ARNT-Like 1 (BMAL1)-BirA∗ knock-in (BMAL1-BioID) mouse. This study found that feeding a 0.5% biotin diet for 7 days induced protein biotinylation in the brain, heart, testis, and liver of BMAL1-BioID mice without adverse effects on spermatogenesis. Additional information regarding biotin administration *in vivo* is provided in the examples described later.

## PL Approaches Used in Neurobiology

**Fig. 2:**
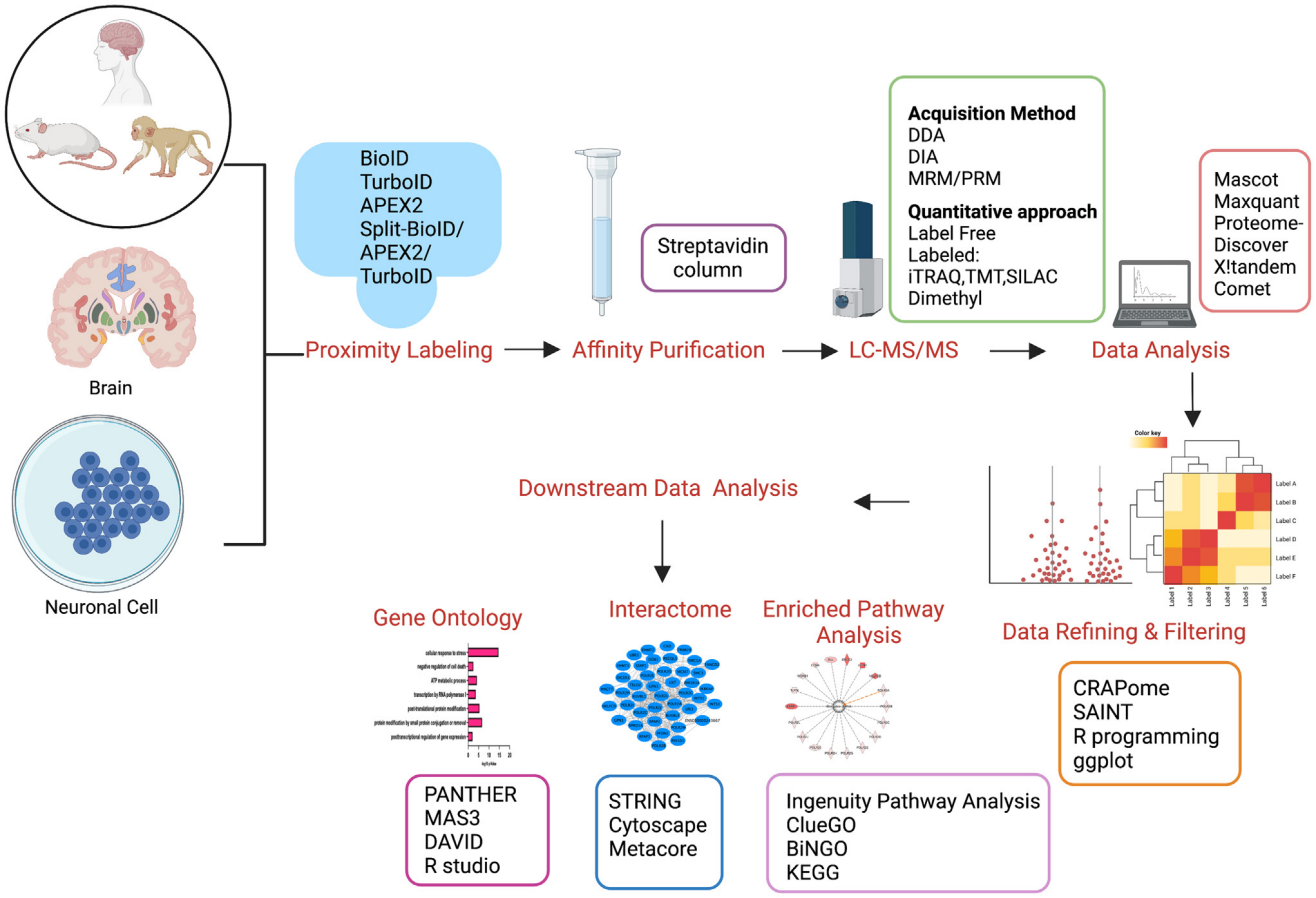
**A proteomic workflow for mapping PPIs in the CNS.** Different regions or specific cell types from brain and neuronal cells in culture can be used for PL. Biotinylated proteins from each sample are enriched and analyzed *via* quantitative MS. Identified interactors are subjected to data refining and filtering to identify significant PPIs. High-confidence proteins are subjected to extensive bioinformatics analysis to identify gene ontology, protein interactome, and enriched pathway analysis to improve understanding of the functional significance of the PPI. CNS, central nervous system; MS, mass spectrometry; PL, proximity labeling; PPI, protein–protein interaction.

The CNS is comprised of morphologically and functionally diverse intermixed cell types and subcellular compartments with distinct proteomes. The different cell types are highly interconnected and utilize specific interactions in a temporal and spatial manner to perform their biological functions (83). Thus, different types of neurons throughout the brain form numerous short-range and long-range synaptic connections that mediate neurotransmission and regulate sensory and behavioral functions. PPIs mediate or regulate every aspect of neuronal cell behavior including cell-to-cell communication, neurogenesis, synaptogenesis, and apoptosis (84). It is also increasingly recognized that PPINs play key roles in accurate information processing, cognition, memory, and reflex behaviors that govern neural communication that can be disrupted in neurodevelopmental disorders (NDDs) (85). Alternatively, PPIN dysregulation can lead to protein aggregation, neuronal stress and dysfunction, and cell death that is associated with various neurodegenerative diseases (84, 86). Therefore, knowledge of the protein interactomes in both healthy and diseased brain offers the potential to advance mechanistic understanding of normal neuronal function as well as the causal basis of neurological disorders. The plethora of PL techniques discussed previously provide an entirely new approach to characterize the proteomes of neuronal and glial cell types and subcellular compartments, allow identification of specific PPIs, and elucidate the dynamics of protein associations in normal brain as well as in a wide range of disease models. In what is a rapidly growing area of research, the results of recent neurobiological studies that used PL are discussed later and summarized in Table 3. An outline of a typical workflow used in many of these studies, which incorporates the methods discussed in sections “PL—general principles, PL enzymes, Biotin ligase–based PL, APEX-based PL, HRP-based PL, Split-PL, Biotinylation by antibody recognition, MS data analysis, and *In Vivo* applications of BioID,” is shown in Figure 2.

**Table 3:** Summary of PL studies that have been coupled to MS-based proteomic analyses to identify and characterize neurobiology-related PPIs

S. no.:	Tissue/cells	PL approach	MS method	Target protein	Clinical condition/classification	Reference
Cell type specific/subcellular						
1	Mice VM dopamine neurons	*Ex vivo*/APEX2	Data-independent acquisition	APEX2-NES	Midbrain subcellular proteomics, cell type specific	(88)
2	Mouse dorsal striatum SPN subtypes	APEX2 *ex vivo*	TMT	Cre-dependent expression of APEX2 in nucleus (H2B fusion), cytosol (NES), or plasma membrane (membrane anchor LCK sequence)	Striatal subcellular, D1 or D2 SPNs, cell type specific	(89)
3	Mouse cortex, hippocampus, striatum/thalamus, pons/medulla, and cerebellum	TurboID	Data-dependent acquisition (DDA) with high-field asymmetric waveform ion mobility spectrometry (FAIMS Pro)	Viral or Cre-dependent expression of TurboID in Ca^2+^/calmodulin-activated protein kinase 2A–expressing neurons	Cell type–specific proteomics	(90)
Synaptic proteins (presynaptic/postsynaptic and synaptic cleft)						
4	*C. elegans*	*In vivo*/TurboID	DDA	ELKS/RAB6-interacting/CAST family member 2	Presynaptic proteins	(92)
5	Rat primary cortical neurons	*In vitro*/HRP	Isobaric tags for relative and absolute quantification (iTRAQ)	Glutamatergic excitatory synaptic proteins (Lrrtm1 and Lrrtm2) and GABAergic inhibitory synaptic proteins (Slitrk3 and Nlgn2)	Excitatory and inhibitory synaptic cleft proteins	(54)
6	Rat primary cortical neurons	*In vitro*/HRP	DDA	Synaptic cell adhesion protein 1/SynCAM1-HRP	Synaptic cleft	(93)
7	Mouse cortex	iBioID	DDA	PSD protein 95 (excitatory synapses) or gephyrin (inhibitory synapses)	Inhibitory and excitatory PSD, synapses	(94)
8	CD1 juvenile mouse brain (P21)	Split-TurboID	DDA	Neuronal-specific promoter (hsyn1) and astrocyte-specific promoter (GfaABC1D) fused to N-terminal and C-terminal TurboID fragments	Astrocyte–neuron signaling, cell–cell interaction	(96)
Axonal growth cone/dendritic spine proteins						
9	Rat primary hippocampal neurons	*In vitro*/BioID	DDA	NF186, nuclear distribution element-like 1 (NDEL1), tripartite motif–containing protein 46 (Trim46)	Axonal initial segment	(97)
10	Mouse cortical cells (*in vivo*)	BioID-2	DDA	Synaptopodin	Dendritic spines, spine apparatus, synaptic proteins	(100)
Lysosomal proteins and endolysosomal pathway						
11	human induced pluripotent stem cell (iPSC)–derived neurons	*In vitro*/APEX	DDA	LAMP1-APEX	Neuronal lysosomal proteins	(101)
12	HEK293 cells	BioID	DDA	TMEM16K	Endolysosomal pathway, spinocerebellar ataxia, endosomal retrograde transport	(102)
Dopamine and glutamate transporter proteins						
13	HT22 neuroblastoma cell lines	*In vitro*/BioID	DDA	DAT	Striatal dopamine transport, neurotransmission, and signal transduction	(103)
14	H22 hippocampal cell line	*In vitro*/BioID	DDA	DAT and glutamate transporter (GLT-1)	Neurotransmission and signal transduction	(104)
Signaling receptors and kinases						
15	SH-SY5Y human neuroblastoma cell line, rat cortical neurons	*In vitro*/photoTurboID	DDA	Mitochondrial–ER lumen–localizing sequence	PTMs, phosphorylation, and signaling	(107)
16	*Drosophila* larval brain	*In vivo*/TurboID and miniTurbo	TMT	Alk	Kinase signaling	(108)
17	HEK293 cells	BioID	DDA	Ephrin-B2 (EphB2)	Cell-surface receptor signaling, neurodevelopment	(109)
NDDs/neurodifferentiation						
18	O9-1 mouse cranial neural crest cell line	*In vitro*/BioID	DDA	Twist Family BHLH Transcription Factor 1 (TWIST1)	Neurodevelopment/differentiation	(111)
19	AD293 cell (HEK cells)	BioID	DDA	TLK2	Mental retardation autosomal dominant 57, neurodevelopment/differentiation	(113)
20	Mouse retinal progenitor cells	*In vitro*/BioID	DDA	Zinc finger transcription factor (Casz1)	Neurodevelopment/differentiation	(114)
21	Mice primary cortical neuronal cells	*In vitro*/BioID	DDA	PARP6	Dendrite morphogenesis, neurodevelopment	(115)
22	Mouse primary cortical neuronal cells	*In vitro*/BioID	DDA	TRIM9 and TRIM67	Neuronal differentiation	(116)
23	*Drosophila* olfactory PNs	*In situ*/HRP	TMT	Rat CD-2	Neurodevelopment, neurodifferentiation	(117)
24	Mouse hippocampus and cortex	iBioID	DDA	Wave-related protein (WRP)-FBAR domain in dendritic filopodia	Synaptogenesis, excitatory synapses, and neurodevelopment	(118)
25	Rat primary hippocampal neurons	*In vitro*/BioID2	DDA	MeCP2	NDDs, Rett syndrome	(119)
Neurodegenerative disorders						
26	Rat primary cortical neurons	*In vitro*/APEX-2	iTRAQ	α-synuclein	PD synucleinopathies	(121)
27	Human neuroblastoma SH-SY5Y cells	*In vitro*/BioID	DDA	α-Synuclein WT or A53T	PD synucleinopathies	(122)
28	Human postmortem tissue—substantia nigra, striatum, cortex	*In situ/*BAR	DDA	Synuclein 1 and phosphorylated synuclein (PSER129)	PD synucleinopathies	(123)
29	Human iPSC–derived glutamatergic neurons	*In vitro*/APEX-2	DDA	N or C terminus truncated Tau	Taupathies, neurodegenerative disorders, AD, and FTD	(126)
30	HT-22 cells (immortalized rat hippocampal cell line)	*In vitro*/BioID	DDA	Beta-site amyloid precursor protein cleaving enzyme 1 (BACE1)	Neurodegenerative disorder and AD	(128)
31	Neuro-2a (N2a) cells	*In vitro*/BirA∗ biotin ligase	DDA	Ataxin-1 protein	Neurodegenerative disease and nuclear transport	(129)
32	Mouse N2a neuroblastoma cells	*In vitro*/BioID	DDA	TDP-43	ALS/FTD, neurodegenerative disorders, cytonucleoplasmic transport	(130)
33	HEK293T	BioID	DDA	Cyclin F	ALS/FTD, cytonucleoplasmic transport	(131)
34	HEK293T cells (American Type Culture Collection CRL-11268)	BioID	diaPASEF	Dipeptide repeat (DPR) proteins detected in the brains of FTD/ALS patients with C9orf72 repeat expansions (c9FTD/ALS)	ALS/FTD, cytonucleoplasmic transport	(132)

### Cell Type–Specific/Subcellular Proteomes

A major challenge in neuroproteomic studies comes from the intermixed nature of many different types of neurons, and their complex axonal and dendritic projections, as well as several types of glia (87). PL can provide spatially resolved, cell type– and subcellular compartment–specific proteome information that is important for understanding neural cell type functions (42). Recently, Hobson *et al.* (88) used APEX2 PL to explore the somatodendritic and axonal proteomes of midbrain dopaminergic neurons. APEX2 fused with a nuclear export sequence (NES) was expressed through injection of conditional adeno-associated virus (AAV) vectors into the ventral midbrain (VM) of DAT^IRES-cre^ mice (Cre recombinase under the transcriptional control of the endogenous dopamine transporter [DAT] promotor). PL was carried out in acute brain slices incubated in oxygenated artificial cerebrospinal fluid (CSF) in the presence of biotin–phenol and H_2_O_2_. Proteomic analysis using a data-independent acquisition approach of streptavidin bead immunoprecipitates from each of the individually dissected regions (VM, medial forebrain bundle, and striatum) of labeled slices showed that each was enriched with region-specific proteins (*i.e.*, 1449 proteins for VM, 702 for medial forebrain bundle, and 1840 for striatum), thus underlining the ability of this technique to identify subcellular cell type–specific proteomes. As part of the validation strategy of cell type enrichment, proteomic data were compared with single-cell RNA-Seq for VM and striatum to generate a high-confidence dataset. To further study the presynaptic proteome of dopaminergic axons, APEX2 labeling was also carried out on synaptosomes prepared from the striatum of DAT^IRES-Cre^ mice expressing APEX2-NES. These analyses identified 1348 proteins that included presynaptic proteins involved in synaptic vesicle trafficking and fusion (SV2A-C, CSPα, synaptogyrin 1–3, synaptophysin, synapsin-1, RAB3A-C, and VAMP-2), and SNARE protein complex (syntaxin 1A/B, SNAP25). A significant number (*i.e.*, 15) of proteins were identified in striatal dopaminergic axons that were related to Parkinson’s disease (PD linked genes) and that might have roles in PD pathophysiology (88).

In another recent study, APEX2 coupled to *ex vivo* analysis was also used to identify differences in cell type–specific subcellular proteomes of two classes of intermingled spiny projection neurons (SPNs), called direct and indirect pathway SPNs, that are the targets of dopamine axons in dorsal striatum of mouse brain (89). Subcellular PL was achieved by expressing Cre-dependent APEX variants in Drd1^Cre^ (direct pathway SPNs) or A2a^Cre^ (indirect pathway SPNs) mouse lines with well-defined sorting motifs that localize to the nucleus (H2B fusion, H2B), cytosol (NES), or plasma membrane (membrane anchor LCK sequence, LCK). This study used an *ex vivo* biotinylation strategy where acutely dissected brain slices (250 μm) were initially incubated in carbogenated artificial CSF supplemented with biotin–phenol, and later biotinylation was induced by H_2_O_2_. A minimum labeling boundary length was established by using a click chemistry–compatible APEX substrate called propargyl tyramide. Tandem mass tag–based quantitative MS and subsequent differential expression analysis between different subcellular APEX variants identified proteins that were specifically enriched in their respective subcellular locales (nucleus, cytosol, or membrane). Subcellular PL in cell types with highly overlapping dendritic architecture and electrophysiological properties was able to differentiate cell type– and subcellular compartment–specific activity-dependent proteome changes (89).

In another recent study, a cell type–specific *in vivo* PL approach that should be applicable to many different neuronal cell types expressed TurboID constructs (*Rosa26*^*TurboID*^) in specific neuronal cells using a Cre-lox genetic strategy (90). After 4 weeks of AAV construct expression, biotin was supplemented to mice through water for 2 weeks. Pan neuronal TurboID expression by bilateral stereotactical injection into hippocampus identified 2143 differentially expressed (*i.e.*, *p* ≤ 0.05, ≥2-fold enrichment) proteins as compared with the controls. These proteins were found to be enriched for proteins involved in neuron projection and synapse organization. This study also examined the proximal proteome of Ca^2+^/calmodulin-activated protein kinase 2A–expressing neurons in different brain regions (cortex, hippocampus, striatum/thalamus, pons/medulla, and cerebellum) in a transgenic mouse by breeding Rosa26^TurboID/wt^ with Ca^2+^/calmodulin-activated protein kinase 2A-Cre^Ert2^ mice. Across all brain regions, 1245 proteins were identified. Principal component analyses of the cell type–specific *in vivo* biotinylation of proteins data identified distinct clusters of proteins (based on the brain region) with 83 signature proteins from cortex/hippocampus, whereas cerebellum contained 138, pons/medulla contained 128, and striatum/thalamus contained 6 proteins. Thus, this study was able to identify distinct neural interactomes in multiple brain regions (90).

### Synaptic Proteins (Presynaptic/Postsynaptic and Synaptic Cleft)

Identifying the spatiotemporal arrangement of PPIs of the multiprotein complexes in synaptic structures should help to increase our understanding of the aberrant synaptic plasticity and synaptic transmission associated with synaptopathies (91). Recently, TurboID was used to identify tissue-specific PPIs in muscles, intestine, hypodermis, and neurons of *Caenorhabditis elegans in vivo* (92). To determine if this approach has sufficient sensitivity to identify PPIs in a specific type of neuron, TurboID-mNeonGreen was transgenically expressed in the Amphid neurons with finger-like AFD thermosensory neurons in the head of *C. elegans*. Compared with nontransgenic animals and pan-neuronal TurboID constructs, 111 AFD-specific proteins were identified that included guanylyl cyclase-8, guanylyl cyclase-18, and the homeobox transcription factor TTX-1 (92). This study also identified specific interactors of the presynaptic protein, ELKS-1, an ortholog of human ERC2 (ELKS/RAB6-interacting/CAST family member 2), by using a CRISPR/Cas9-mediated TurboID approach in *C. elegans*. Several known ELKS-1-interacting active zone proteins, as well as previously uncharacterized synaptic proteins, were identified. In this study, an optimum level of proximal protein biotinylation was achieved by using biotin-auxotrophic *E. coli* as a food source before biotinylation and a 2 h incubation with exogenous biotin (92).

In a PL-based study, HRP-fused constructs of two known proteins each from glutamatergic excitatory synapses (Lrrtm1 and Lrrtm2) or GABAergic inhibitory synapses (Slitrk3 and Nlgn2) were used to differentially identify excitatory and inhibitory synaptic proteomes (54). A chemically synthesized membrane-impermeable variant of biotin–phenol (BxxP) with a long and polar polyamide linker limited the proximal protein biotinylation to the targeted neuronal surface compared with a nonspecific HRP-TM, which biotinylated the entire neuronal surface. This study used technical improvements such as a 1% SDS lysis step (with boiling) to remove detergent-insoluble PSD proteins from the biotinylated cleft proteome. After differential expression analysis with HRP-TM, there were 199 and 42 unique proteins identified in excitatory and inhibitory synapses, respectively, with 20 proteins common to the two proteomes. Gene Ontology analysis showed that 84% of the excitatory proteome and 90% of the inhibitory proteome were synaptic proteins, and of the remaining 33 proteins, termed as “synaptic orphans,” a majority of the proteins were validated as synaptic proteins using immunofluorescence and immunoblotting (54). Finally, follow-up studies implicated CD200 and Mdga2 in synaptic cleft function.

PL of excitatory synaptic cleft proteins in rat primary cortical neurons was also carried out using synaptic cell adhesion protein 1 (SynCAM1) fused to HRP as a reporter. SynCAM1 localizes exclusively to excitatory synapses during development and in mature synapses (93). A membrane-impermeable biotin–phenol derivative (biotin-AEEA-phenol) was used to ensure that biotinylation would be restricted to synaptic cleft as opposed to intracellular proteins. Ratiometric comparison of membrane-HRP *versus* SynCAM1-HRP targeted PL and multiple filters were used to specifically identify 39 synaptic cleft candidate proteins that were found in multiple biological replicates. These included several known synaptic cleft proteins such as Neuroligin-3, Neurexin-1, Latrophilin-3, Contactin-1, Kilon, Hapln1, and Noelin-1. In addition, several novel cleft proteins were identified, and one of these, receptor-type tyrosine–protein phosphatase zeta, was successfully validated (93). This study demonstrates that PL can be used to determine the molecular composition and specific PPIs in compartmentalized structures such as synapses that are not readily accessible to traditional biochemical techniques.

PL was used to target bait proteins specific to postsynaptic excitatory and inhibitory synapses by *in vivo* expression of AAV constructs in the cortex of P0 mouse pups containing either PSD protein 95 (PSD-95) (excitatory synapses) or gephyrin, collybistin, and inhibitory synaptic protein 1 (inhibitory synapses), each fused with BirA (94). Biotin was subcutaneously injected for 7 consecutive days following 35 days of AAV viral construct expression. Previously uncharacterized proteins, such as inhibitory synaptic protein 1 and 2, named as Insyn1 and Insyn2, respectively, were identified in pilot studies with BirA-gephyrin. Proteins were considered significantly (*p* < 0.05) enriched in the bait fraction if their average BirA-fusion protein levels were ≥2-fold greater than in the BirA-only fractions. Using these criteria, the 121 PSD proteins that were identified included >95% of the proteins that had been previously identified by traditional PSD biochemical fractionation and MS/MS analyses (*i.e.*, 116 of 121 proteins). The PSD proteins included glutamate receptors, scaffolding proteins, and signaling proteins in excitatory synaptic complexes. The 181 proteins in the inhibitory synapse dataset included nearly all previously reported inhibitory synapse proteins as well as a large number of new proteins. These included trafficking, cytoskeletal regulatory, and integral membrane proteins as well as several protein kinases and phosphatases. Interestingly, 15% (27 of 181) of these inhibitory synapse proteins have been shown to be involved in seizure susceptibility in humans or mice or other brain disorders such as intellectual disabilities. Of the 17 proteins that were common to the PSD and inhibitory synapse datasets, about half are signaling proteins (94). In follow-up functional studies, CRISPR-based depletion of Insyn1 resulted in decreased postsynaptic inhibitory sites, reduced miniature inhibitory postsynaptic currents, and increased excitability in the hippocampus that may result from the disruption of interactions between other inhibitory postsynaptic proteins such as gephyrin and dystrophin (94). Taken together, these results demonstrate the ability of PL to identify functionally relevant PPIs in the PSD and that the inhibitory postsynaptic proteome is more complex than was previously appreciated.

As discussed previously, split PL enzymes such as HRP that are coexpressed as complementary fragments can be reactivated when they approach each other as a result of a PPI between the respective baits (95). To initially assess feasibility, the HRP fragments (sHRPa and sHRPb) were fused respectively to neurexin and neuroligin, which bind each other across the synaptic cleft, and were shown to reconstitute in HEK293T cells (95). To determine if sHRP could also detect synapses *in vivo*, Martell *et al.* investigated the mouse visual system. Amacrine cells in the retina form synapses on retinal ganglion cells (RGCs) that in turn extend axons through the optic nerve to “retinorecipient” regions in the brain, including the superior colliculus (SC). sHRPa-neurexin was introduced into the RGCs by AAV and sHRPb–neuroligin into the SC by electroporation. Four weeks later, mice were perfused with heme-containing media, and colliculi were removed, incubated with heme, fixed, sectioned, and stained for sHRP activity and expression of the sHRP fragments. Punctate sites of sHRP activity were present in regions where sHRPa-positive RGC terminals apposed sHRPb-positive SC cells. Puncta were also localized within the retinorecipient layer within which retinocollicular synapses are known to form (95).

Later, the split technique was modified and used to identify protein interactions in the extracellular cleft between astrocytes and neurons. Since the molecular mechanisms that drive astrocyte–synapse adhesion and that enable astrocytes to control synapse formation and function are largely unknown, a combination of TurboID and Split-TurboID PL was used to identify PPIs at astrocyte–neuron junctions (96). The Split-TurboID enzyme constructs (N-TurboID and C-TurboID) that were expressed on the extracellular surfaces of astrocytes and neurons recovered their activity when astrocytes and neurons were in close proximity. Biotin was subcutaneously administered (24 mg/kg) for 7 consecutive days after 3 weeks of viral construct expression. This study identified 118 high-confidence proteins in the tripartite synapse (a combination of presynaptic and postsynaptic neuronal and perisynaptic astrocytic processes) proteome and characterized proteins that play key role(s) in astrocyte–neuron signaling. CRISPR-based depletion of one of these bridging proteins, neuronal cell adhesion molecule, showed that homophilic binding of astrocytic and neuronal cell adhesion molecule restricts the growth of astrocyte processes into the neuropil and controls the organization of inhibitory synaptic specializations (96). These results significantly increase our knowledge of how astrocytes interface with neurons and control GABAergic synapse formation and function.

### Axonal Growth Cones/Dendritic Spines

Although the mechanisms of Na^+^ and K^+^ channel clustering by axon initial segments (AISs) have been delineated, the molecular mechanisms that stabilize the AIS and that control neuronal polarity are not well understood. To gain additional insight into AIS functions, Hamdan *et al.* (97) used a multiplexed PL approach that involved targeting different bait proteins to provide more comprehensive coverage of the AIS proteome in hippocampal neurons. Because of the large size of the AIS, which is located within the first 30 μm of the axon (98), and the limited (*i.e.*, approximately 10 nm (32, 40)) BirA∗ labeling range, it was necessary to fuse BirA∗ to multiple proteins from different regions of the AIS. Hence, BirA∗ was fused to proteins from the intracellular membrane (neurofascin-186 [NF186]), cytoplasmic (Ndel1), and microtubule (Trim46) compartments to comprehensively profile the AIS interactome and to also provide conceptual insights into AIS molecular organization, mechanisms of AIS stability, and polarized trafficking in neurons. The control used for NF186-BirA∗ was a chimera with a deletion of five amino acids (NF186ΔFIGQY-BirA∗) that does not localize to the AIS. In addition, BirA∗ linked to the N terminus (BirA∗-Ndel1C) or C terminus (Ndel1C-BirA∗) of Ndel1 enabled identification of those cytoplasmic proteins in AIS that were nonproximal (>10 nm) to NF186. Since the N- and C-terminal Ndel1-BirA∗ are separated by a largely unstructured 179 amino acid residue sequence, the contour length of Ndel1C (3.4–4.0 Å/residue) suggests the two chimeras may map regions as much as ∼60 to 70 nm apart. The numbers of candidate proteins that were identified in each proximity proteome were 73 for NF186-BirA∗, 55 for Ndel1C-BirA∗, and 77 for Trim46-BirA∗. Nine proteins were common to all three proteomes, and a total of 164 unique proteins were identified in the entire AIS proteome—with many not being previously reported as AIS proteins. In addition to providing a valuable resource for AIS molecular organization and function, this study demonstrates how PL can be used to profile the PPIs in larger biological structures by multiplexing the labeling with spatially separated target proteins.

Dendritic spines are postsynaptic compartments found at excitatory synapses that organize neurotransmitter signaling and contain structural elements including the spine apparatus that is part of the ER (99). Recently, the protein interactome of synaptopodin, a major spine apparatus protein, was investigated using an iBioID-based PL approach (100). Synaptopodin-BioID2 or Shank3∗-BioID2 (a PSD protein used as a control) was expressed in cerebral cortices of neonatal mice using AAV/9 for 5 weeks, after which biotin (24 mg/kg) was injected subcutaneously for 7 consecutive days. Differential expression analysis identified 140 enriched proteins in the synaptopodin interactome. These included proteins involved in calcium storage, signaling, lipid metabolism, and transfer, which are all consistent with the known association of synaptopodin with the ER. In addition, there were a large number of scaffold and actin-related proteins. Additional studies confirmed that three identified proteins (*i.e.*, Pdlim7, Magi1, Magi2) are colocalized with synaptopodin in dendritic spines. Further validation studies suggest that Pdlim7, which is an actin-binding protein not previously identified in spines, acts as an anchor to stabilize the assembly of SA proteins (100).

### Lysosomal Proteins and Endolysosomal Pathway

The proteomic microenvironment of the neuronal endolysosomal network was explored (101) by knocking the APEX gene into the endogenous locus of lysosomal-associated membrane protein 1 (LAMP1) in human-induced pluripotent stem cell–derived neurons. Nonspecifically labeled proteins were identified by comparing LAMP1-APEX with the control, cytosol localized NES-APEX. Normalizing the PL proteomic data to the most abundant endogenously biotinylated protein (PCCA) provided an effective way to reduce variations between biological replicates and different APEX labeling experiments and probes. PL identified several stable lysosomal membrane proteins (*e.g.*, LAMP1, LAMP2, LAMTORs, PIP4P1, PIP4P2, V-ATPases) as well as transient lysosomal-interacting proteins involved in lysosomal transport, mobility, and signaling pathways. For example, the identified Rab GTPases and SNARE proteins play crucial roles in endolysosomal trafficking, autophagy, and lysosome biogenesis. In addition, multiple components of the V-ATPase complex and key proteins in the mammalian target of rapamycin complex 1 pathway also were identified (101).

The crucial role of the lipid scramblase TMEM16K in endolysosomal retrograde transport and its potential association with an autosomal recessive form of progressive neurodegenerative disease, spinocerebellar ataxia (SCAR10), was explored by PL using TMEM16K-BioID in HEK293 cells (102). The TMEM16K interactome contained multiple proteins that function at ER endosomal contact sites, including VAPA, VAPB, SNX1, SNX2, Rab7A, and PTP1B, suggesting that TMEM16K acts at or near these membrane contact sites. Network analyses showed that endosomal transport, in particular endosomal retrograde trafficking, was a major cluster in the TMEM16K interaction network. Other studies found that the Rab7 GTPase, a TMEM16K proximal protein that is present in ER–endosomal contact sites, colocalizes with TMEM16K and that loss of TMEM16K function results in impaired endosomal retrograde trafficking and neuromuscular function, one of the symptoms of SCAR10 (102).

### Dopamine and Glutamate Transporter Proteins

With the goal of increasing our understanding of DAT function, a BioID-based PL approach was employed to identify potential protein interactors of DAT in the HT22 neuroblastoma cell line (103). The 21 proximal biotinylated proteins included integral plasma membrane proteins such as 4F2 cell-surface antigen heavy chain (Slc3a2), membrane-associated progesterone receptor component (PGRMC2), neuronal membrane glycoprotein (M6a), and cytoplasmic proteins such as F-box/LRR-repeat protein 2 (FBXL2) and phosphatase phosphatidylinositol 3,4,5-trisphosphate 5-phosphatase 2 (Inppl1/SHIP2). Colocalization of DAT with these interacting proteins in dendritic spines, axonal tracts of primary neurons, and the functional studies that were carried out in this study help to confirm the role of these proteins in DAT-mediated neurotransmission (103). A previous study from the same group reported that a common interacting protein of DAT and glutamate transporter (GLT1), K^+^ channel Kv7.3, plays a major role in transporter function and that the polarization effect of K^+^ channels is affected by the transporters in return (104). These reports indicate that PL is an excellent choice for studying PPIs of transmembrane proteins, which are challenging to study using traditional AP–MS approaches.

### Signaling, Kinases, and Post-Translational Modifications

Post-translational modifications (PTMs), particularly phosphorylation and dephosphorylation, play key roles in many aspects of neuronal function (105). PTMs have been found to be strategically located to allow for the proper orchestration of PPIs, resulting in distinct functional roles in molecular processes (106). Indeed, PTM-carrying proteins appear to engage in more PPIs and are positioned in more central network locations than non-PTM proteins (106). Thus, alteration in PTMs resulting from regulation of cellular signaling pathways will likely alter PPIs. Importantly, MS has made it possible to identify thousands of PTMs in single experiments. However, as summarized by Liu *et al.* (107), existing PL enzymes are not readily applicable to the analysis of dynamic PTM changes at the subcellular level for several reasons; for example, (1) the APEX requirement for H_2_O_2_ (∼1 mM) would affect cellular metabolism and protein PTMs; (2) cellular expression of TurboID would result in biotinylation of endogenous proteins, which may alter cellular processes and give rise to cytotoxicity; or (3) the biotin starvation commonly used in BioID/TurboID assays to reduce background labeling could result in substantial stress to cellular homeostasis that alters normal protein PTM patterns. To address these challenges, Liu *et al.* (107) designed and then integrated a photoactivated TurboID biotin ligase with an orthogonal phosphorylation enrichment strategy, termed subcellular-specific uncaging-assisted biotinylation and mapping of phosphoproteome (SubMAPP), for assessing the phosphorylation dynamics of subcellular proteomes in living systems. The SubMAPP strategy was shown to be applicable to neuronal cell culture models and cultured rat primary neurons. Finally, to circumvent the limitation of using photoTurbo in deep tissue or intact animals, a chemical-activatable Turbo was developed that extends the SubMAPP strategy to living mice (107).

The protein interactome of anaplastic lymphoma kinase (Alk), which is an evolutionary conserved receptor tyrosine kinase implicated in neuronal function, was explored using three different variants of PL tags (Alk BirA∗, miniTurbo, and Turbo) expressed in *Drosophila* larval (third instar) brain using a CRISPR/Cas9 gene editing approach (108). Comparative analysis showed that miniTurboID, which identified 142 proteins, and TurboID, which identified 140 proteins, outperformed the first-generation BioID that identified only two proteins. The increased labeling with miniTurboID and TurboID may result from the improved ability of these next-generation BirA∗ enzymes to label at the comparatively low temperature of 25 °C that is the standard growth temperature for *Drosophila*. This study also suggests that miniTurboID and TurboID permit efficient biotin labeling of proximal proteins at early stages of embryogenesis without external biotin supplementation. Additional studies validated several Alk interactors and identified the protein tyrosine phosphatase corkscrew as a modulator of Alk signaling (108).

To identify interactors of an activated ephrin (Eph) tyrosine kinase receptor and to better understand its relation to Eph clustering and endocytic sorting, a PL study was carried out in HEK293 cells that inducibly expressed EphB2-BirA∗ and that had been stimulated by exposure to Eph-B2 for 6 h (109). Included among the BirA∗-enriched proteins were several known EphB2-binding proteins such as Abelson kinase (ABL2) and Nck adaptor proteins (NCK1 and NCK2) (109). In addition, another protein interactor, His domain phosphotyrosine phosphatase (HD-PTP), which is a known endosomal sorting complex required for transport adaptor protein with trafficking functions, had not previously been shown to be involved in Eph signaling, Additional studies confirmed that HD-PTP binds to EphB2 and that HD-PTP plays a crucial role in EphB2-mediated Eph-B–EphB signaling in axonal growth cone and spinal neuronal development. This study thus established a functional link between Eph signaling and endosomal sorting complex required for transport accessory proteins that revealed their novel role in promoting cell surface receptor signaling. Finally, identification of interactomes in Eph-evoked Eph signaling, which occurs on the scale of minutes, suggest that even though biotinylation of EphB2-proximal proteins proceeded for 6 h, it was still successful in identifying presumably relatively short-lived PPIs (109).

### NDDs/Neurodifferentiation

PPIs play a major role in the development of the nervous system (110). The protein interactome necessary for normal development of cranial neural crest cells (NCCs) was explored by using Twist Family BHLH Transcription Factor 1 (TWIST1) as an anchor protein in a BioID-based PL approach (111). NCCs are a multipotent and migratory cell population that contribute to the creation of a variety of tissues (craniofacial skeleton, connective tissues, melanocytes, neurons, and glia) in the developing embryo (112). BioID followed by MS analysis of a cranial NCC cell line, O9-1, identified 140 TWIST1 interactors that included four known proteins. The remaining interactors were further screened using network propagation analytics, which is an approach that has been used for predicting gene function and for identifying important protein modules. This predictive approach identified a TWIST1-chromatin regulatory module (TWIST1-CRM) in NCC. Perturbation of four core members of the TWIST1-CRM (*i.e.*, TWIST1, CHD7, CHD8, and WHSC1) in cell models and mouse embryos revealed that TWIST-CRM is required for normal differentiation of NCCs and craniofacial tissues (111).

BioID-based PL was used to increase our understanding of the molecular basis of NDDs, such as intellectual disability and mental retardation autosomal dominant 57. The proximal interactome of missense mutations (D551G and S617L) of Tousled-like kinase 2 (TLK2), previously shown to be associated with these disorders, was compared with WT TLK2 by expressing the respective BioID-fused tags in AD293 cells (113). PL interactome analysis indicated that both missense mutants resulted in reduced interactions with several replication fork and autism spectrum disorder–related proteins including DNA repair protein RAD50 and YEATS2, whereas interactions with JMJD1C, BRD4, CCNK, NACC1, MSANTD2, and CHD8 were reduced with the TLK2-S617L mutant. In addition, both mutants showed increased interactions with other autism spectrum disorder–related proteins including NFIA and NFIX, as well as with ZNF148 and PAPOLG, that are associated with NDD. The reduced interactions with several replication fork proteins (RAD50 and BRD4) suggest that chromatin maintenance defects are involved in the pathogenesis of the TLK2 variants (113).

PPIs involved in the temporal identity transitions that occur in retinal development have been explored by PL studies that were carried out on the Casz1 zinc finger transcription factor (Casz1) that had been tagged with BirA in retinal progenitor cells (114). Core subunits of the NuRD nucleosome remodeling and deacetylase complex comprised five (*i.e.*, Chd4, Gatad2a/b, and Mta1/2) of the eight proteins that were in the Casz1 BioID interactome. The remaining proteins were the linker histones Hist1h1b/d (H1.3 and H1.5) and histone 2B. Linker histones play key roles in heterochromatin compaction, which agrees with the observed enrichment of Casz1 and NuRD proteins on the margins of chromocenters. In follow-up studies, the authors showed that both NuRD and the polycomb repressor complexes are required for Casz1 to promote rod fate and to suppress gliogenesis (114).

Although poly-(ADP-ribose) polymerase 6 (PARP6) is known to regulate dendritic morphogenesis, its function in the nervous system is not well understood. To gain further insight into PARP6 function, a BioID-based PL study was carried out in rat primary cortical neuronal cells (115). Four of the five PARP6 interactors identified were microtubule-associated proteins (*i.e.*, MAP2, MAP1B, EB2, and Tau) that play important functional roles in regulating the microtubule cytoskeleton in neurons, especially during early development. These results suggest that PARP6 may play a role in regulating the neuronal microtubule cytoskeleton during development (115).

In another study of cortical neuron morphogenesis, the interactome of two proteins (TRIM9 and TRIM67) in the E3 ubiquitin ligases family, which play a major role in axonal growth cone projection, dendritic arborization, and spine density in adult-born neurons, was explored using a BioID-based PL approach (116). A Myc-BirA∗ tag was fused to TRIM9 or TRIM67, which lacked the ligase domain to minimize enrichment of biotinylated ubiquitin. Chimeric proteins were expressed in mouse primary cortical neurons through herpes simplex virus transduction. The PL approach using TRIM9 and TRIM67 identified 149 and 151 interacting proteins, respectively (false discovery rate [FDR] values < 0.25), with ∼60% overlap between the two interactomes. Gene Ontology analyses showed that TRIM9 protein interactors are involved in neuronal arborization, microtubule organization, and synapse structure, whereas TRIM67 interactors are involved in synapse localization, cytoskeletal polymerization, and miRNA-mediated translation inhibition. This study suggests that the interactomes of these two ubiquitin ligases coordinate cytoskeletal dynamics and plasma membrane expansion and thereby facilitate neuronal growth and the establishment of functional neuronal networks (116).

The cell surface proteome and its differential expression during the developmental stages of olfactory projection neurons (PNs) was explored using PL in intact *Drosophila* brain (117). PN surface proteins were profiled by expressing an HRP-fused rat CD-2 transmembrane protein at two time points; (a) 36 h after puparium formation, when developing PNs send out their dendrites and axons to establish synaptic connections and (b) 5 days after eclosion into adults, when mature PNs are actively processing olfactory information. *In situ* biotinylation of intact brain using a membrane-impermeable biotin-xx-phenol (Bxx*P*) substrate identified 403 and 561 proteins, respectively, in developing mature PN surface proteomes. Of the 712 proteins in the PN surface proteomes, 252 proteins were common to the developing and mature proteomes, whereas 460 were specific to either developing or mature PNs. These data suggest profound differences between the developing and mature PN surface proteomes. While the most significant biological functions of the developing proteome were processes involved in neuronal projection development, the mature surface proteome included categories covering ion channels, receptors, and transporters. This PL technique also identified several unconventional proteins that had not previously been associated with neuronal development. The involvement of these novel proteins with neurodevelopment was confirmed by selective RNAi knockdown experiments (117). In this study, the use of cell-surface targeted HRP and a biotin substrate impermeable to the plasma membrane allowed specific biotinylation of PN surface proteins. A similar approach could be used to target cell surface proteins in growing axonal tip and medium spiny neurons, and so on.

An iBioID study identified nascent synaptic proteins in cortex and hippocampus by taking advantage of the dendritic filopodial targeting of the FBAR domain of the actin binding wave-related protein (118). Adenovirus carrying fused iBioID (AAV BirA) probes were injected intracranially into hippocampus and cortex of mouse pups at postnatal day 0 (P0). The pregnant mice were then injected with 24 mg/kg biotin at E17.5 for 3 days prior to delivering a litter of pups. The pups were also administered daily subcutaneous doses of biotin from P0 to P5 to ensure robust *in vivo* biotinylation. This study identified 60 proteins within nascent dendritic spines that included various excitatory synaptic proteins like latrophilins, Wiskott–Aldrich syndrome protein family member 1 (WAVE1), and actin-related protein 2/3 (Arp2/3 or CARMIL3), which all play a critical role in generating branched actin filaments required for excitatory postsynaptic development. The specific depletion of CARMIL3 resulted in a significant decrease in synaptic spine density and AMPAR-dependent excitatory synaptic neurotransmission (118). Thus, iBioID provided a highly efficient approach to identify PPIs within developing excitatory postsynapses that are difficult to identify through traditional biochemical approaches and that could also be useful in identifying alterations in protein interactions involved in neurodevelopmental abnormalities *in vivo*.

The role of methyl-CpG binding protein 2 (MeCP2) and its interacting partners in the pathophysiology of NDDs such as Rett syndrome (RTT) and MECP2 duplication syndrome has been explored using a BioID-based PL approach in rat primary neurons (119). Compared with the BioID constructs of the negative controls (MeCP2^R111G^ and MECP2^ΔNLS^) that disrupt DNA binding and nuclear localization of MeCP2, respectively, the BioID WT MeCP2 interactome had five proteins (PHF14, TCF20, CHAMP1, RPS11, and SF3B1). TCF20 and PHF14 function as subunits of a putative chromatin-binding complex that includes two other subunits, RAI1 and HMG20A. Co-IP studies confirmed these two proximal interactions by identifying the critical interacting domains of MeCP2 specific to TCF20 and PHF14. In addition, a missense mutation in PHF14 (PHF14-C322G) was identified in a patient with RTT-like, and other symptoms, which disrupts the PHF14 interaction with MeCP2 and TCF20 (119). Taken together, these data help to demonstrate the critically important role of the MeCP2–TCF20 complex for normal brain function.

### Neurodegenerative Disorders

PL has increasingly been used to identify the interactomes of various proteins that have been implicated in the pathophysiological mechanisms of NDs. APEX2 PL was used to identify the protein interactome of alpha-synuclein (α-syn), whose nucleotide polymorphism, misfolding, and intraneuronal accumulation is associated with various synucleinopathies (120). Viral-mediated expression of α-syn-APEX2 in rat primary cortical neurons showed a neurite-specific punctate labeling pattern. While α-syn-APEX2 was punctate in neurites, NES-APEX2 displayed diffuse cytoplasmic and neurite labeling (121). Biotinylated proteins from α-syn-APEX2, NES-APEX2, and APEX2-negative neurons were quantified using isobaric tags for relative and absolute quantification. Filtering the >2000 proteins that were identified by MS by comparing the α-syn-APEX2 condition *versus* the NES-APEX2 control identified 225 enriched proteins proximal to α-syn in cortical neurons. These proteins are involved in various biological functions such as membrane trafficking (Ras-related protein Rab-6A, synaptojanin), RNA binding (polyadenylate-binding protein 1, TIA1 cytotoxic granule-associated RNA-binding protein), and include signaling proteins and phosphatases (calcineurin, serine/threonine-protein phosphatase PP1-alpha catalytic subunit). The α-syn interactome was validated using complementary techniques that included membrane Y2H and co-IP studies (121). Another study used BioID to compare the protein interactomes of α-syn with a variant α-syn that has a point mutation (A53T) linked to familial PD. This study, which was carried out in SH-SY5Y human neuroblastoma cells expressing the BirA∗-fused Myc-tagged WT or mutant (A53T) α-syn, identified transcriptional adapter 2-alpha (TADA2a) as a novel binding partner of α-syn (122). TADA2a is a component of the p300/CBP-associated factor that is a histone acetyltransferase that plays an important role in the remodeling of chromatin and the regulation of gene expression. It has been shown to preferentially catalyze acetylation of the epsilon-amino group of lysine 14 in histone H3. Compared with α-syn WT, the A53T α-syn was more preferentially localized in the nucleus. The resulting increased binding of α-syn A53T with TADA2a resulted in decreased levels of acetylation of histone H3, which may be involved in α-syn-mediated neurotoxicity in the pathophysiological mechanism of PD (122).

Recently, an *in situ* BAR PL approach was used to compare the protein interactomes of α-syn with a pathological phosphorylated form of α-syn (PSER129) that is a marker of Lewy body pathology in human postmortem tissue obtained from individuals diagnosed with synucleinopathies (SYN) that include PD and dementia with Lewy bodies (123). The BAR strategy was used to biotinylate proximal proteins in midbrain and striatum of postmortem tissue of two individuals diagnosed with PD, one with dementia with Lewy bodies and three healthy controls. This study had three experimental conditions, BAR-SYN1 (total α-syn), BAR-PSER129, and control sample (without primary antibody). Differential expression analysis showed that the BAR-PSER129 identified 261 proteins that were significantly enriched in the α-syn as compared with the healthy control samples. Further bioinformatic analysis of the differentially expressed proteins showed that the most significant Kyoto Encylopedia of Genes and Genomes pathway was PD, and the most significantly enriched Gene Ontology compartments were the vesicle, extracellular vesicle, extracellular exosome, and extracellular organelle. This study provides evidence that PL approaches can be used to identify PPIs of proteins with PTMs present in insoluble aggregates like Lewy bodies and amyloid plaques in human formalin-fixed brain tissues (123). Notably, the BAR strategy overcomes the issues of protein fusion, but the approach requires a highly specific antibody and could be sensitive to artifacts caused by fixation and protein modifications because of postmortem time intervals (124).

Tau is a microtubule-binding protein whose aberrant PTM has been implicated in Alzheimer’s disease (AD) and other tauopathies (125). A recent study used APEX2 PL and isolation of biotin-labeled peptides to identify the protein interactomes of Tau in human-induced pluripotent stem cell–derived glutamatergic neurons (126). This study identified distinct protein interactions specific to the N- or C-terminal regions of Tau, which are separated by 63 to 70 nm, by expressing WT 2N4R Tau tagged at the N or C terminus with APEX2. Comparative expression analysis of biotinylated proteins from N-APEX Tau and C-APEX Tau samples *versus* APEX-α-tubulin samples identified 246 Tau-interacting proteins, of which 136 were enriched in both N-APEX Tau and C-APEX Tau samples. These proteins included known interactors such as alpha-synuclein, MAP1A, and protein phosphatase 5. In addition, the Tau interactome was enriched for SNARE complex proteins (*e.g.*, SNAP25, SNAP29, Munc18). Domain-specific analysis of biotinylation sites on SNARE complex and synaptic vesicle proteins suggested that in human neurons Tau is tightly connected to the cytosolic membrane surface of synaptic vesicles *via* assembled SNARE complexes at vesicle fusion sites (126). Differential expression analysis of the N- and C-terminal interactomes of Tau revealed that 45% of all interactions were specific to either N-APEX or C-APEX Tau samples. The N-terminal interactome included proteins that regulate dendritic spines, postsynaptic organization, proteasome-dependent protein degradation, and vesicle transport. The C-terminal interactome included proteins that mediate cellular stress response and presynaptic vesicle fusion (126).

Anomalous production of amyloid beta peptides by the sequential cleavage of amyloid precursor protein leads to the formation of amyloid plaques, one of the hallmarks of AD pathology (127). The protein interactome of beta-site amyloid precursor protein cleaving enzyme 1 (BACE1) was identified using BioID PL (128). In this study, mouse hippocampal–derived cells (HT22) were transfected with BACE1-BirA tag and lysed after 24 or 48 h. LC–MS/MS analyses of tryptic digests of the streptavidin-bound fractions identified 33 and 27 proteins in the 24 h and 48 h (FDR < 0.1) samples, respectively (128). The most over-represented protein at both time points was the membrane-associated progesterone receptor component 2. This study provides the first data supporting a potential role for BACE1 in hormone signaling. Although this study identified several putative BACE1 protein interactors, the lack of identification of known AD-associated BACE1 substrates in the interactome, the absence of independent validation experiments, and the less stringent criteria used for protein identification (*i.e.*, FDR < 0.1) all suggest that additional studies are needed to confirm these preliminary findings.

The polyglutamine (polyQ) tract form of ataxin-1 drives disease progression in spinocerebellar ataxia type 1 (SCA1). Although known to form distinctive intranuclear bodies, the cellular pathways and processes that polyQ-ataxin-1 influences remain poorly understood. In conjunction with pull-down studies, PL was used to determine the protein composition of the ataxin-1 interactome by conjugating BirA∗ biotin ligase to the 85Q form of ataxin-1 in Neuro-2a cells (129). The 85Q form of ataxin-1 was primarily localized in the nucleus and was present in the nuclear bodies that are a characteristic feature in individuals with SCA1. Differential expression analysis confirmed the previously identified ataxin-1 interactor protein, Capicua transcriptional repressor, along with various proteins involved in the nuclear transport pathway (*i.e.*, Ran signaling). Further targeted analyses suggest that increased levels of polyQ [85Q] ataxin-1 disrupt multiple essential nuclear protein trafficking pathways, increasing our understanding of the pathophysiology of SCA1 (129).

Although the cytoplasmic mislocalization and aggregation of TAR DNA-binding protein-43 (TDP-43) is a common histopathological feature of amyotrophic lateral sclerosis (ALS) and frontotemporal dementia disease (FTD) spectrum, little is known about the composition of these aggregates and their involvement in the disease process. An *in vitro* BioID approach determined that detergent-insoluble TDP-43 cytoplasmic aggregates are enriched in NPC and nucleocytoplasmic transport proteins (130). The MS analysis of biotinylated proteins from Neuro-2a cells transfected with myc-BirA∗-tagged human TDP-43 (myc-BirA∗-TDP-43) or TDP-C-terminal fragment (myc-BirA∗-TDP-CTF), which is a major component of insoluble cytoplasmic aggregates in ALS/FTD, identified 254 proteins and 389 proteins in the interactomes of TDP-43 and TDP-CTF, respectively. While the TDP-43 interactome was enriched for proteins involved in mRNA processing, the TDP-CTF-associated proteome was enriched for proteins involved in intracellular transport with a major subset of these proteins being involved in the nucleocytoplasmic transport pathway. This study suggests that cytoplasmic mislocalization/aggregation of TDP-43 disrupts NPCs and nucleocytoplasmic transport and provides a common disease mechanism for ALS and FTD (130).

Similarly, BioID PL approaches were used to identify the protein interactomes for cyclin F, which is a substrate recognition component of an E3 ubiquitin ligase (131), and dipeptide repeat (132), proteins that have been shown to be involved in the pathology of FTD and ALS. Both studies expressed the respective BioID constructs in HEK293 cells and identified the interactome networks. Notably, the study by Rayner *et al.* (131) linked ALS/FTD-causing mutations in cyclin F to pathological features of ALS/FTD that included defective protein degradation systems and the accumulation of a protein, SFPQ, involved in RNA processing and metabolism. The study by Liu *et al.* (132) determined that molecular chaperones play a central role in the pathogenesis of ALS/FTD and uncovered a possible novel therapeutic approach.

## Conclusions and Future CNS Perspectives

Proximity-dependent biotinylation with engineered enzymes, in combination with MS-based quantitative proteomics, has emerged as a powerful method to dissect molecular interactions and localizations of endogenous proteins. Currently, there is “no ideal” method for all situations, rather, each PL technique has its own strengths and caveats. All but one (126) of the PL studies mentioned in the neurobiology section (see “PL Approaches Used in Neurobiology” section) used streptavidin-based beads to enrich the biotinylated proteins, but recently, peptide-level enrichment using antibiotin antibody (133) or neutravidin bead enrichment (134) of proteolytic digests of biotinylated proteins, have shown promising results (126), and is likely to reveal more detailed information about PPIs. Irrespective of the PL method used, it is critical to use controls and quantitative methods to filter out nonspecific false-positive hits. Secondary validation is also required. In the case of large datasets from, for example, specific cell type studies, proteomic data can be compared with single-cell RNA-Seq data (88). Immunohistochemical methods are required to confirm both cell type and subcellular location. Importantly, since PL cannot distinguish between direct binding *versus* the close physical proximity of two proteins, PPI studies should be expanded and interactors further validated and characterized by carrying out *in vitro* binding, Y2H, IP, or other affinity techniques. Finally, proximal proteins identified for a specific bait protein could be further validated using selective CRISPR/Cas9-mediated knockdown of specific protein interactors (135, 136), as was done in some of the examples discussed (118).

PL methods are particularly useful for probing transient or relatively weak PPIs in neuronal samples that are lost following cell disruption and interactions that involve low-abundance and membrane-localized proteins. In this respect, PL methods are ideal for generating high-resolution PPI maps of complex proteomes found in the normal and diseased brain. Based on a rapidly expanding array of genetic and viral tools, cell type–specific PL techniques can be used, for example, to identify PPIs in a variety of neuronal and glial subtypes, as well as in their dendritic and axonal subcompartments. PL also can be used to target dynamic proteins that are translocating between organelles (*e.g.*, cytonuclear translocation). Cell–cell junction structures like synapses contain a variety of biologically important proteins that are difficult to purify without contamination from other adjacent structures. PL achieved by targeting specific bait proteins can be used to overcome these technical limitations. As a result, PL of proteins specific to excitatory or inhibitory synapses may help to identify PPIs that result in maladaptive neurotransmission. Synaptic reorganization and alterations in synaptic plasticity because of psychostimulants and other clinical conditions can be explored using PL techniques. Proteomic mapping of the interface of neurons and glia using split PL methods can be used to decipher protein interactions and signaling between different cell types in the brain.

Development of new and modified PL enzymes and optimization of currently available PL methods will no doubt further enhance their applicability. For example, in a recent study, a split BioID construct termed Cal-ID was designed to be responsive to cytosolic levels of Ca^2+^ (137). Experiments in cultured neurons identified specific PPIs near plasma membrane Ca^2+^ ATPases. Thus, function-dependent activation of PL constructs could be used to identify protein interactors in response to neurotransmitters or ion influx/efflux in neuronal systems. To investigate dynamic changes in neuronal physiology at the proteome level, future research could combine PL with optical and electrophysiological slice manipulations. Another focus area may be secreted vesicular bodies, such as exosomes, apoptotic bodies, and stress vesicles, that are involved in signaling between neurons, astrocytes, microglia, and Schwann cells. Targeting specific proteins that are known to be secreted in these vesicles should enable biotinylation of their associated proteomes. Such studies, including their isolation from biofluids like CSF or plasma, may help to understand how these vesicles are specifically targeted to different cell types and elucidate their role in the pathophysiological mechanisms of various clinical conditions. Integrating PPI analyses with protein expression, phenotype, and other datasets will no doubt provide powerful opportunities for elucidating neuroproteomic functions.

## Conflict of interest

The authors declare no competing interests.
